# The role of microRNAs in glucocorticoid action

**DOI:** 10.1074/jbc.R117.000366

**Published:** 2018-01-04

**Authors:** Sally A. Clayton, Simon W. Jones, Mariola Kurowska-Stolarska, Andrew R. Clark

**Affiliations:** From the ‡ Institute of Inflammation and Ageing, College of Medical and Dental Sciences, University of Birmingham, Birmingham B15 2WB,; the ¶ Institute of Infection, Immunity and Inflammation, College of Medical, Veterinary and Life Sciences, University of Glasgow, Glasgow G12 8TA, Scotland, and; the § Arthritis Research UK Rheumatoid Arthritis Pathogenesis Centre of Excellence (RACE), Glasgow, Birmingham, and Newcastle Universities, Glasgow G12 8TA, Scotland, United Kingdom

**Keywords:** epigenetics, microRNA (miRNA), glucocorticoid, glucocorticoid receptor, apoptosis, inflammation, epigenetics

## Abstract

Glucocorticoids (GCs) are steroids with profound anti-inflammatory and immunomodulatory activities. Synthetic GCs are widely used for managing chronic inflammatory and autoimmune conditions, as immunosuppressants in transplantation, and as anti-tumor agents in certain hematological cancers. However, prolonged GC exposure can cause adverse effects. A detailed understanding of GCs' mechanisms of action may enable harnessing of their desirable actions while minimizing harmful effects. Here, we review the impact on the GC biology of microRNAs, small non-coding RNAs that post-transcriptionally regulate gene expression. Emerging evidence indicates that microRNAs modulate GC production by the adrenal glands and the cells' responses to GCs. Furthermore, GCs influence cell proliferation, survival, and function at least in part by regulating microRNA expression. We propose that the beneficial effects of GCs may be enhanced through combination with reagents targeting specific microRNAs.

## Introduction

Both the fields of glucocorticoid (GC)^3^ biology and microRNA biology are too vast and complex to survey in detail here. Instead, we will give brief overviews of both areas, drawing attention to excellent recent reviews, before focusing on the functional interactions between GCs and microRNAs, which have not been comprehensively reviewed elsewhere to our knowledge.

## Glucocorticoids

GCs are a group of steroid hormones that have varied roles in development, homeostasis, and circadian regulation of physiological processes (1–5). Among many effects, they suppress inflammatory gene expression in several cell types and promote apoptosis of some hematopoietic cells. These anti-inflammatory and/or immunosuppressive effects are the basis of the widespread use of synthetic glucocorticoids to prevent transplant rejection and graft *versus* host disease and to treat chronic inflammatory disorders and hematological malignancies (6). However, severe, even life-threatening side effects can arise from prolonged exposure to synthetic glucocorticoids, as well as from elevated production of endogenous glucocorticoids, as seen in the rare endocrine cancers known as Cushing's disease. Common side effects include osteoporosis, hypertension, mood disorders, muscle and skin atrophy, and increased susceptibility to infection. Although these side effects are often said to limit the clinical utility of GCs, prolonged treatment with GCs is far from uncommon, particularly among the elderly. Since the discovery of GCs by Philip Hench and others in the 1940s, the uncoupling of their desirable anti-inflammatory or immunosuppressive effects from their harmful side effects has been pursued with little success (7).

**Figure 1.**
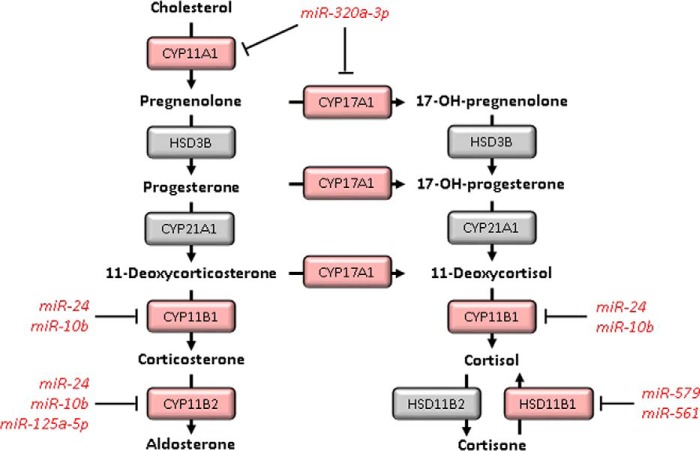
**Regulation of GC biosynthesis by miRNAs.** The endogenous human glucocorticoid cortisol and the mineralocorticoid aldosterone are synthesized in the adrenal cortex from cholesterol. miRNAs have been reported to regulate this synthesis by the targeting of cytochrome p450 enzymes.

The production and actions of GCs are subject to tight regulation at several levels. Here, we will briefly mention some of these complex processes and direct interested readers to more comprehensive reviews (2–5). 1) Like the related hormone aldosterone, the GC cortisol is produced from the precursor cholesterol in the adrenal cortex, via sequential enzymatic reactions mediated by hydroxysteroid dehydrogenases (HSD family) and cytochrome P450 oxidases (CYP family) (Fig. 1). Cortisol biosynthesis is regulated in circadian fashion by a hormonal relay system known as the hypothalamic–pituitary–adrenal (HPA) axis. The hypothalamus produces corticotropin-releasing hormone, which acts upon the anterior pituitary to induce production of adrenocorticotropic hormone (ACTH). ACTH stimulates the adrenal gland to synthesize and release GCs, principally cortisol in humans. Cortisol also acts on both the hypothalamus and pituitary glands to suppress the HPA axis and limit its own production via negative feedback. 2) Local GC availability is regulated by interconversion between the active GC cortisol and inactive cortisone (8). The conversion of cortisol to cortisone is chiefly mediated by 11β-hydroxysteroid dehydrogenase 2 (11βHSD2), whereas the reverse reaction is chiefly performed by 11βHSD1. 11βHSD2 is most strongly expressed in the kidney, where it protects the aldosterone receptor from inappropriate activation by GCs. Expression of 11βHSD1 in many cells and tissues promotes GC responsiveness. 3) The GC receptor (GR) is encoded by the *Nr3c1* gene in mice (*NR3C1* in humans). Alternative transcription initiation and splicing generate a variety of mRNA species (3). These mRNA isoforms, in addition to alternative use of translational initiation sites, generate GR protein products that differ in function. 4) GR is a transcription factor, which is thought to exert most of its effects at the level of transcriptional control (although non-genomic effects are also increasingly recognized). In the absence of ligand, GR is held in the cytoplasm in complex with chaperone proteins, including FKBP5 (FK506-binding protein 5), which maintain its ligand–receptive conformation but also mask its nuclear localization signal. GCs are lipophilic and readily diffuse across the plasma membrane. Binding to GR causes the release of GR from its chaperone complex and translocation to the nucleus. 5) GR can bind directly to DNA, most often as a homodimer recognizing a palindromic element with consensus half-site sequence AGAACA. It can also bind to DNA as a monomer recognizing a single half-site, or it can be recruited to DNA indirectly, via interaction with various other DNA-binding proteins. Whichever mode of binding to DNA is used, GR may regulate transcription either positively or negatively, depending on cellular and chromatin context. Higher order chromatin structure and the presence of DNA-bound partner proteins dictate where GR is recruited and which genes it is able to regulate. Most of the GC regulome is cell type-specific, and relatively few genes are universally GC-responsive. 6) GR is extensively phosphorylated within its N-terminal half (1). A few of its phosphorylation sites have been functionally characterized and shown to modulate GR function, either globally or in a target gene-specific manner. Other post-translational modifications such as SUMOylation have also been described.

## MicroRNAs

MicroRNAs (miRNAs) are short RNA species, generally 19–22 nucleotides in length, which mediate post-transcriptional down-regulation of protein expression (9). This occurs by sequence-specific recognition of seed sequences predominantly in the 3′UTR of target mRNAs. Regulation via 5′UTRs or even open reading frames may also occur, but it is poorly understood and believed to be rare (10, 11). The majority of mammalian miRNA/mRNA interactions result in mRNA degradation, brought about by transcript deadenylation through recruitment of the CCR4–NOT deadenylation complex (9, 12). Alternatively, translational repression can occur due to inhibition of translational initiation (13), in at least some cases as a consequence of mRNA deadenylation. Up to 60% of the mammalian transcriptome is estimated to be subject to regulation by miRNAs (9, 14).

**Figure 2.**
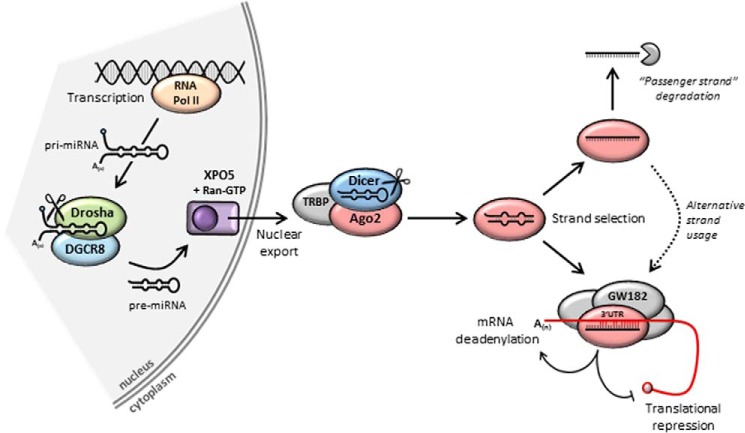
**miRNA biogenesis and function.** miRNAs are transcribed from the genome to create primary (*pri-*)miRNAs. The first round of cleavage occurs in the nucleus, carried out by the RNase III enzyme Drosha and associated factor Pasha/DGCR8, resulting in the shorter precursor (*pre-*)miRNA. Nuclear export is mediated by Exportin 5 (XPO5) and Ran-GTP. The second cleavage is carried out in the cytoplasm by the RNase III enzyme Dicer as part of a protein complex that produces the mature miRNA. Strand selection results in release of one of the two miRNA strands, which is degraded. The retained strand is loaded onto Argonaute protein (*e.g.* Ago2) and forms RISC along with other proteins such as GW182. Silencing can occur by promoting deadenylation and mRNA degradation or translational repression.

The miRNAs are generally transcribed from the genome by RNA polymerase II (Fig. 2). They can be expressed from within protein–coding genes, most often from their introns or 3′UTRs; from miRNA clusters, where multiple miRNAs are generated from a single precursor transcript; or as single miRNAs within non-coding regions. They are synthesized as initial extended RNA transcripts, called primary miRNAs. The initial transcript undergoes its first round of processing by the RNase III enzyme Drosha and associated protein Pasha/DGCR8 (DiGeorge syndrome critical region 8), which cleave it into the 70-nt precursor miRNA. This is transported out of the nucleus and undergoes a second round of cleavage, performed by the RNase III enzyme Dicer, resulting in the mature miRNA as a duplex around 21 nts in length (15). The miRNA functions as part of an RNA-induced silencing complex (RISC), in which the Argonaute family of proteins plays a major role. The mature miRNA is loaded onto Argonaute (Ago1–4 in mammals), and the passenger strand is removed and degraded, resulting in the final active complex containing a one-stranded miRNA species complementary to a portion of the target gene 3′UTR. The regulation of strand selection in miRNA maturation is not yet fully understood. There is increasing evidence that either strand of the miRNA duplex can be selected as a guide for Ago proteins and independently regulate expression of distinct targets (16, 17).

## Regulation of glucocorticoid availability by miRNAs

Global inhibition of miRNA production by knockdown of the processing enzyme Dicer increased the production of cortisol by adrenocortical cells, implying that steroidogenesis is negatively regulated by miRNAs (18, 19). Among mRNAs whose expression significantly increased following Dicer knockdown were *CYP11A1*, *CYP11B1*, and *CYP17A1*, which encode cytochrome oxidase enzymes involved in cortisol biosynthesis or interconversion between aldosterone and cortisol biosynthetic pathways (Fig. 1). miR-24 and miR-10b were implicated as negative regulators of *CYP11B1* (19, 20), and miR-320a-3p as a negative regulator of both *CYP11A1* and *CYP17A1* (18). Although individual miRNAs exert relatively little effect on cortisol biosynthesis, coordinated changes of miRNA abundance may have a more striking impact, for example in the context of hypoxia or adenocarcinoma (18, 20). Local GC availability can also be regulated via effects of miRNAs on expression of 11β-HSD enzymes. miR-579 and miR-561 were identified as negative regulators of 11β-HSD1 expression (21). In theory, differences in expression of these miRNAs could have an impact on metabolic side effects of GC excess (8, 22).

## Regulation of the glucocorticoid receptor by miRNAs

GCs regulate many aspects of neuronal development and function, having particularly important roles in adaptation to stress. Expression of GR is high in the hypothalamus and other regions of the brain forming the primitive limbic system, which is central to stress responses (23). Increased GR expression improves resilience to at least some forms of experimental stress (24). Physiologically, miRNA-mediated modulation of GR expression is thought to play a role in fine-tuning responses to GCs during development of the central nervous system (25). Pathophysiologically, prolonged exposure to stress or elevated GC levels can cause long-lasting reprogramming of neuronal GC responses, which can result in neuropsychiatric disorders such as depression in later life. Such reprogramming at critical developmental stages *in utero* may contribute to the transgenerational effects of antenatal GC exposure (23, 26).

The 3′UTR of the canonical *Nr3c1*/*NR3C1* transcript contains a number of well-conserved binding sites for miRNAs (27), of which we will initially focus on miR-124-3p. This neuronally expressed miRNA targets the *Nr3c1* 3′UTR to down-regulate GR expression (28–32). It can be induced by GCs (28, 32–34), suggesting that it may contribute to negative-feedback regulation of GC sensitivity. In rats (31, 34) and mice (32), elevated miR-124-3p levels in the brain were associated with decreased GR levels and GC sensitivity and increased depression-like symptoms. Furthermore, treatment of mice with an miR-124-3p antagonist (antagomir) reduced depression-related behaviors (32). Levels of miR-124-3p were reportedly elevated in peripheral blood of patients with severe depression (35, 36). Collectively, these findings suggest that miR-124-3p may be both a biomarker and therapeutic target for psychiatric disorders related to dysfunction of the HPA axis. It should be noted that several other putative targets of miR-124 have been identified (37), and miR-124 was reported to influence GR function indirectly via effects on phosphodiesterase 4B (38) or 11βHSD1 (34).

ACTH treatment of mice increased adrenal gland expression of several miRNAs, including miR-142-3p (although, curiously, not miR-124-3p) (39). A number of these miRNAs were demonstrated to target the *Nr3c1* 3′UTR and reduce expression of GR. As suggested above, miRNA-mediated fine-tuning of GR levels may contribute to desensitization in the face of sustained activation of the HPA axis, preventing consequences such as GC-induced atrophy of the adrenal glands. Other miRNAs that have been implicated in the post-transcriptional control of GR expression include miR-101a and miR-18a (31, 39, 40). Increased expression of miR-18a and decreased expression of GR were found in the paraventricular nuclei of Fischer 344 rats, which fail to demonstrate adaptive responses to repeated stress and display increases in stress-related disorders (40).

An interesting concept is that differential exon usage at the *Nr3c1* locus may generate transcripts that differ in their susceptibility to miRNA-mediated regulation. Differential splicing at the last intron gives rise to an mRNA isoform that encodes GRβ but lacks binding sites for miR-124-3p, miR-142-3p, and others (28). The GRβ protein isoform is transcriptionally inactive and is believed to function as a dominant-negative inhibitor of the canonical GRα isoform (41). Therefore, either changes of mRNA splicing or miRNA expression could result in an increased GRβ/GRα ratio and an impairment in GC sensitivity. Cell type-specific alternative promoter usage at the *Nr3c1* locus generates transcripts containing variant, non-coding first exons. It has been speculated but not yet established that these mRNA isoforms differ in sensitivity to miRNA-mediated regulation (42). Targeting of 5′UTRs by miRNAs differs from the canonical mechanism but has been demonstrated in other contexts (10).

High-dose GCs are often used to treat leukemias such as multiple myeloma (MM) and acute lymphoblastic leukemia (ALL) because of their capacity to impair cell division or promote apoptosis. Resistance to these effects is a common and serious clinical problem. miRNA-mediated suppression of GR has been identified as a possible causative event in the escape of leukemias from GC therapy (27, 43). Expression of miR-124-3p was elevated in ALL patients that showed poor response to GC therapy and was accompanied by reduced expression of GR (44). Introduction of miR-124-3p to a GC-sensitive cell line *in vitro* attenuated the anti-proliferative and pro-apoptotic effects of GC treatment (44). High expression of miR-142-3p in ALL was also correlated with poor prognosis (45). This miRNA was shown to inhibit pro-apoptotic effects of GCs by targeting both GC and cyclic AMP-signaling pathways, which cooperate to regulate T cell apoptosis (45, 46). It appears that escape from the inhibition of tumor growth may depend on miRNAs that normally function to attenuate GC responses in the context of sustained HPA axis dysregulation and/or GC excess. The high concentrations of GCs used in cancer treatment may generate selective pressure for cells expressing high levels of *NR3C1*-targeting miRNAs or directly promote increases in expression of those miRNAs. The miRNA-mediated regulation of GR expression has also been implicated in the loss of GC sensitivity in MM. The *NR3C1*-targeting miR-130b was more strongly expressed in a GC-insensitive MM line, and its introduction to a GC-sensitive line impaired cellular response to GC treatment, including the induction of apoptosis (47). However, the use of an antagomir to knock down miR-130b expression in the GC-insensitive line was not sufficient to restore GC sensitivity, suggesting that additional factors (miRNA or other) must contribute to GC resistance.

GR function is positively and negatively regulated via phosphorylation (1), and it can be indirectly modulated at this level by miRNAs. For example, miR-9 targets a regulatory subunit of protein phosphatase 2A (PP2A), leading to an increase in activity of the PP2A substrate c-Jun N-terminal kinase 1 (JNK1) and enhanced phosphorylation of GR (48). Phosphorylation by JNK1 impairs GR function by inhibiting nuclear translocation of the receptor. An miR-9 antagonist restored GC responsiveness in experimental models of GC-resistant asthma (48). Another indirect mechanism for modulation of GR function involves miR-511-5p-mediated control of the expression of the chaperone protein FKBP5, alleviating GR repression by this chaperone and contributing to neuronal differentiation (49). miR-433 was reported to dampen GR activity indirectly by suppressing its ligand-induced translocation to the nucleus, but a detailed mechanism underlying this miRNA effect has yet to be described (50).

## Role of miRNAs in leukocyte responses to glucocorticoids

Several miRNAs have been implicated in positive or negative regulation of inflammatory and immune responses (51, 52), and such mechanisms may be subject to modulation by GCs. The best characterized example is miR-155, which is generated from the precursor transcript B cell integration cluster (BIC) (53). Integration of the avian leukosis virus at the BIC locus in chickens increased the expression of BIC and its product miR-155, with oncogenic consequences. Increased levels of miR-155 have subsequently been described in many types of cancer, leading to its identification as one of the first oncogenic miRNAs (oncomirs). In myeloid cells, the expression of miR-155 is induced by engagement of pattern recognition receptors, in a manner that is at least partly dependent on nuclear factor κB (NF-κB) (54, 55). It promotes inflammatory responses by inhibiting the expression of negative regulators, including suppressor of cytokine signaling 1 (SOCS1), a negative regulator of type 1 cytokine and Toll-like receptors; Src homology 2-containing inositol phosphatase 1 (SHIP1), a negative regulator of the TLR/PI3K/Akt pathway; and Bcl6, a negative regulator of NF-κB (53). miR-155 is highly expressed in both fibroblasts and macrophages of the inflamed synovium in rheumatoid arthritis (RA), where it contributes to the elevated expression of chemokines and pro-inflammatory cytokines (56, 57). Mice lacking miR-155 were resistant to synovial inflammation and bone erosion in an experimental model of RA, identifying this miRNA as a critical inflammatory mediator (57). The GC dexamethasone inhibited the lipopolysaccharide (LPS)-induced expression of miR-155 in primary macrophages and macrophage cell lines (54), spleen and liver cells of LPS-injected mice (54, 58), and T lymphocytes of sepsis patients (59). These observations collectively suggest down-regulation of miR-155 is an important anti-inflammatory action of GCs. Dexamethasone-mediated down-regulation of a BIC promoter construct in the macrophage cell line RAW264.7 was dependent on an NF-κB-binding site, although curiously, the mutation of this site did not strongly impair the activation of the promoter by LPS (54). The exact mechanism by which GCs inhibit miR-155 expression requires further clarification. Another important question is whether GCs alter B cell function via changes in expression of miR-155. There is growing evidence for a pathogenic role of miR-155 in Th2-mediated allergic diseases such as asthma (60), which are commonly treated using GCs. However, miR-155 expression was neither elevated in airway cells of patients with mild asthma nor was successful glucocorticoid therapy associated with any change in its expression (61). In fact, no GC-induced changes of miRNA abundance were found in this study or another from the same group (62), in which pulmonary inflammation in mice was induced by LPS inhalation. Either GC-mediated changes of miRNA expression did not contribute strongly to the suppression of inflammation *in vivo*, or the heterogeneity of cell types sampled in lung biopsies concealed cell type-specific GC responses.

miR-511 is generated from the fifth intron of *Mrc1* mRNA, which encodes the C-type mannose receptor CD206. Expression of CD206 is elevated in so-called alternatively-activated or M2 macrophages and can be up-regulated by GCs as well as the M2-inducing cytokines IL-4 and -13 (63, 64). miR-511-5p was shown to target *Tlr4* (Toll-like receptor 4) and *Il12b* (IL-12p40 subunit), contributing to impaired sensitivity to LPS and decreased expression of the classically-activated (M1) macrophage marker IL-12 (64). The SPRET/Ei mouse strain is resistant to TNFα-induced systemic inflammation and displays hyperactivity of the HPA axis. The consequently high levels of endogenous GCs increased the expression of miR-511-5p, which targeted *Tnfrsf1a* mRNA to down-regulate its protein product, the p55 TNF receptor, conferring TNF resistance (63). Thus, changes in levels of miR-511-5p or its opposite strand partner miR-511-3p may have an important impact on myeloid cell differentiation and activation, as a by-product of altered *Mrc1* gene expression (65, 66). Another GC-induced miRNA, miR-98, targeted *Tnfrsf1b* mRNA and reduced expression of its product, the p75 TNF receptor, in T lymphocytes (67). miR-98, like other members of the let-7 miRNA family to which it belongs, is also thought to target the 3′UTR of *Il13* (67, 68). Because IL-13 has a well-established pathogenic role in asthma, miR-98 may contribute to therapeutic effects of GCs in this disease.

Mitogen-activated protein kinase (MAPK) p38 is a critical mediator of inflammatory responses in myeloid and other cells. Its activity is dynamically regulated by a negative feedback loop involving its dephosphorylation and inactivation by dual specificity phosphatase 1 (DUSP1) (69, 70). A number of pro- and anti-inflammatory mediators influence the duration and strength of inflammatory responses by modulating the expression of DUSP1 (70, 71). Notably, GCs cooperate with pro-inflammatory stimuli to enhance and sustain DUSP1 expression and to accelerate the inactivation of p38 MAPK (69, 70). Both direct GR-mediated activation of the *Dusp1* gene (72, 73) and indirect miRNA-mediated effects (74) have been described. Pro-inflammatory stimuli induce the expression of miR-101, which targets the *Dusp1* 3′UTR. GCs inhibit the expression of miR-101, contributing to sustained DUSP1 expression, impaired p38 MAPK activation, and reduced expression of p38 MAPK-dependent inflammatory mediators (74).

GCs influence lymphocyte cell division and apoptosis, effects that can be exploited for the treatment of many hematological malignancies. As reviewed elsewhere (43), tumor-suppressive effects of GCs are now thought to involve changes in expression of miRNAs. The miRNAs that regulate GC responses by altering the expression of GR were discussed above. The miR-17∼92 cluster consists of six miRNAs (miR-17, -18a, -19a, -19b-1, -20a, and 92a-1) generated from a single precursor RNA that is transcribed from chromosome 13 (75). The chromosomal region is duplicated in many human lymphomas, and overexpression from a transgenic construct causes lymphoproliferative disease (76). Targets of the miRNA cluster include the tumor suppressor PTEN (phosphatase and tensin homolog), which regulates inositol phosphate signaling; and Bim, a pro-apoptotic member of the Bcl-2 family of proteins, which collectively regulate cytochrome *c*-mediated apoptosis. Several reports have ascribed therapeutic effects of GCs in lymphoma to the suppression of the miR-17∼92 cluster, and consequent induction of Bim-mediated apoptosis (77–80). Conversely, resistance to GC-induced apoptosis was associated with failure of GCs to down-regulate the miR-17∼92 cluster (77, 81). This miRNA cluster appears to play a well-conserved role in fine-tuning of responses to pro- and anti-apoptotic signals. However, the mechanism of regulation by GCs remains to be identified.

Other GC-regulated miRNAs that may contribute to the regulation of survival have been identified by deep sequencing of leukocytes undergoing GC-induced apoptosis (80, 82) or screening for differentially expressed miRNAs in GC-sensitive and -insensitive cancer cell lines (81, 83). One such differentially expressed miRNA is miR-150-5p, which was up-regulated in a GC-sensitive MM cell line but not in a GC-insensitive cell line. Although overexpression of miR-150-5p in the GC-insensitive line reprised many of the effects of GCs on genes involved in proliferation and survival, it failed to induce apoptosis (81). Additional effectors of the GC response therefore remain to be identified. Conversely, resistance to GC-induced apoptosis in MM has been linked to increased expression of miR-221/222 (84) or miR-125b (83). These miRNAs are likely to exert their effects by impairing cell death pathways, including DNA damage-induced apoptosis driven by TP53 (tumor protein 53). Paradoxically, miR-125b is up-regulated by GCs (83), perhaps a mechanism by which GCs self-limit their pro-apoptotic effects. One implication of these observations is that therapeutic promotion of apoptosis in hematological malignancies could be augmented by supplementing GCs with miRNA antagonists (83, 84). However, successful therapeutic manipulation of miRNA expression is likely to be highly specific to tumor type and mechanism of oncogenesis. This is demonstrated by the comparison of miR-221 expression between cancers. Resistance to GC-induced apoptosis has been attributed to an increase of miR-221 expression in MM (84) but a decrease of miR-221 expression in some forms of ALL (85).

Plasmacytoid dendritic cells (pDCs) are key to the innate immune defense against viral pathogens. They express high levels of type I interferons upon stimulation with agonists that mimic viral infection, such as ligands of Toll-like receptor 9 (TLR9). Such agonists render pDCs resistant to GC-induced apoptosis (86). Treatment of pDCs with GC alone led to up-regulation of several miRNAs, including members of the miR-29 family that can target anti-apoptotic members of the Bcl2 family. Addition of a TLR9 agonist abrogated GC-induced apoptosis, which was restored by transfection of miR-29b or miR-29c mimics (87). Therefore, activation of pDCs impairs their GC-induced apoptosis at least partly by inhibiting the expression of miR-29 family members.

## Role of miRNAs in non-hematopoietic cell responses to glucocorticoids

Bone is continuously remodeled via balanced activities of bone-forming cells (osteoblasts) and bone-resorbing cells (osteoclasts). A common and severe side effect of prolonged exposure to GCs is osteoporosis, resulting from decreased proliferation or increased apoptosis of osteoblasts, combined with increased proliferation or resorptive activity of osteoclasts (88). Treatment of osteoblasts with GC decreased the expression of the miR-17∼92 cluster (89). Both miR-17 and miR-20a inhibit osteoblast expression of receptor activator of NF-κB ligand (RANKL), a factor critical for driving the differentiation and activation of osteoclasts (90). Therefore, decreased miR-17∼92 expression could indirectly favor bone resorption. As described in lymphoma cells, decreased miR-17∼92 expression also promotes Bim-mediated apoptosis of osteoblasts, providing a direct mechanism of GC-induced bone loss (89). Down-regulation of the miR-17∼92 cluster was also reported to contribute to GC-induced apoptosis of chondrocytes, suggesting that this mechanism of control of apoptosis may be widespread (91).

Several miRNAs have been reported to target components of the Wnt (wingless type)-signaling pathway, which fine-tunes bone metabolism (92, 93). miR-199a-5p is up-regulated by GC treatment of osteoblasts and targets two members of the WNT pathway, WNT2 and FZD4, a member of the Frizzled family of plasma membrane WNT receptors (94). However, miR-199a-5p levels were found to be diminished in bone samples from patients with Cushing's disease, a condition of endogenous GC excess (95). The reason for the discrepancy is not known. It could be related to the heterogeneous cellular composition of bone samples studied or an adaptation to prolonged elevation of endogenous GC. miR-320b is also up-regulated by GC treatment of osteoblasts, causing reduction in the expression of β-catenin, the key downstream mediator of Wnt signaling (96). Other candidate miRNA mediators of GC effects on bone were also identified in this study. Prolonged GC treatment of rats decreased the expression in bone of several miRNAs, including miR-29a (97). Retroviral delivery of miR-29a precursor RNA protected rats against GC-induced bone loss, whereas the miR-29a antagonist mimicked the effects of GC. Increased levels of miR-29a were accompanied by increased expression of osteogenic markers such as collagen type 1a1 and the transcription factor Runx2. However, direct targets of miR-29a were not identified. It was suggested that miR-29a could be utilized to prevent bone pathologies associated with long-term GC use. A note of caution is necessary here. Osteoblast-specific deletion of the miRNA-processing enzyme Dicer did not prevent GC-induced inhibition of bone formation (98). The most obvious interpretation is that miRNAs do not play a major role in GC-induced osteoporosis. However, it is also conceivable that global disruption of miRNA biogenesis in osteoblasts has balancing positive and negative effects on bone turnover.

Several studies have reported a broad down-regulation of mature miRNA expression in response to GC treatment. This has been linked to the reduced expression of the miRNA processing enzymes Drosha, Dicer, and Pasha/DGCR8, with a resulting reduction in total miRNA maturation (78, 89, 91). Silencing of Dicer expression by shRNA in lymphocytes enhanced the apoptosis induction by GCs (78). However, the general consequences of a global inhibition of miRNA production are unclear, due to the broad roles of miRNAs and the varied context-specific effects of GCs. The proposed mechanism of miRNA inhibition is also inconsistent with the multiple reports of GC-induced miRNA up-regulation in multiple cell types (67, 80, 95, 96) and the observations of transcriptional inhibition, for example of the miR-17∼92 cluster (79). These studies do, however, highlight the many potential mechanisms by which GCs can alter miRNA abundance, either in a miRNA-specific manner or more broadly.

## Summary

**Figure 3.**
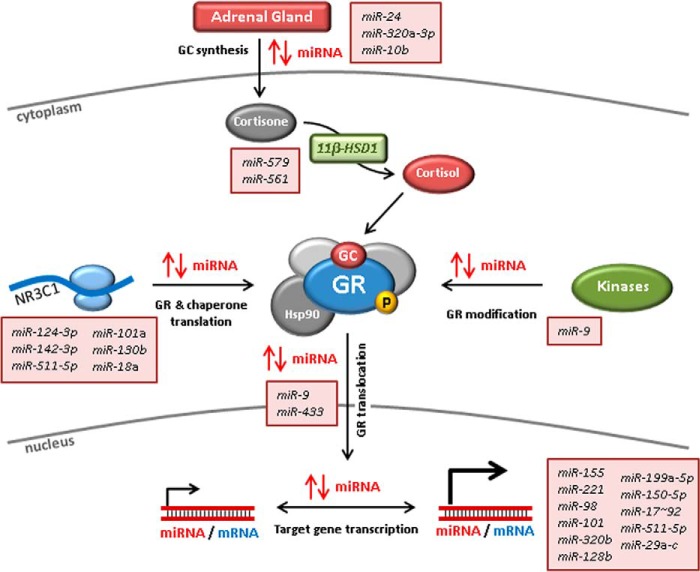
**Impact of miRNAs on GC biology.** miRNAs modulate every aspect of GC biology, including the biosynthesis of GCs in the adrenal cortex, the localized conversion of cortisone to cortisol, the expression of the GC receptor, and its ability to respond to GC. Expression of miRNAs is also positively or negatively regulated by GCs as a means of bringing about changes in cell proliferation, survival, and function.

As miRNAs exert such pervasive effects on biological processes, it is not surprising that they touch on GC action at several points (summarized in Fig. 3). Their touch is often light, in the sense that individual miRNAs alter expression of their targets only modestly. Nevertheless, one miRNA can hit several targets, and one target may be hit by several different miRNAs. Therefore, coherent and coordinated changes of miRNA abundance can affect complex biological processes quite profoundly. Furthermore, functional interactions between miRNAs and target transcripts are based on limited sequence complementarity. Such regulatory complexity creates two sets of major challenges. The first are essentially challenges of methodology and bio-informatics. How are functional interactions to be predicted with confidence, and how are subtle effects of individual miRNAs to be experimentally validated without the danger of confirmation bias? How are miRNA-mediated effects on biological processes to be identified and understood when they involve many-to-many rather than one-to-one interactions? These challenges are increasingly being met through improvements in miRNA target prediction algorithms, systems biological approaches, better methodologies, and more widespread adoption of best-practice experimental controls. The second set of challenges relate to the therapeutic exploitation of knowledge gained. In this context, advances are likely to be most rapid in situations where insensitivity to GCs is a pressing clinical problem, for example in hematological malignancies and GC-resistant asthma. Even where good therapeutic targets can be clearly identified, it remains to be seen whether a mimic or antagonist of a single miRNA species will be sufficient to exert therapeutic effects. If targeting more than one miRNA proves necessary, this will create additional barriers to development, in part because of the problem of predicting and mitigating off-target effects. This field of endeavor is an exciting one, but success is far from certain.

## Footnotes

3 The abbreviations used are:

GC: glucocorticoid
HSD: hydroxysteroid dehydrogenase
ACTH: adrenocorticotropic hormone
GR: GC receptor
ALL: acute lymphoblastic leukemia
TLR: Toll-like receptor
RA: rheumatoid arthritis
LPS: lipopolysaccharide
TNF: tumor necrosis factor
MAPK: mitogen-activated protein kinase
pDC: plasmacytoid dendritic cell
HPA: hypothalamic–pituitary–adrenal
miRNA: microRNA
nt: nucleotide
RISC: RNA-induced silencing complex
MM: multiple myeloma
BIC: B cell integration cluster.
